# Poly[[(4,4′-bipyridine-κ*N*)[μ_3_-(*S*)-2-hy­droxy­butane­dioato-κ^4^ *O* ^1^,*O* ^2^:*O* ^4^:*O* ^4′^]zinc] dihydrate]

**DOI:** 10.1107/S1600536811045788

**Published:** 2011-11-05

**Authors:** Yu-Kun Lu, Jian Liu, Cheng-Lin Diao, Ren-Qing Lü, Yun-Qi Liu

**Affiliations:** a State Key Laboratory of Heavy Oil Processing, College of Science, China University of Petroleum (East China), Qingdao Shandong 266555, People’s Republic of China; b Tingyi (Cayman Islands) Holding Corporation, Tianjin 300457, People’s Republic of China

## Abstract

In the title compound, {[Zn(C_4_H_4_O_5_)(C_10_H_8_N_2_)]·2H_2_O}_*n*_, the Zn^II^ ion displays a distorted tetra­gonal–pyramidal coordination environment with one hy­droxy O and three carboxyl­ate O atoms from three malate anions, and the one remaining position occupied by an N atom from a 4,4′-bipyridine ligand. The pyridine rings of the 4,4′-bipyridine ligand are twisted with respect to each other by a dihedral angle of 35.8 (2)°. The uncoordinated water mol­ecules are linked to the complex mol­ecules by O—H⋯O hydrogen bonds. Each malate anion forms four coordination bonds with three Zn atoms, establishing a layer structure parallel to the *ac* plane. Adjacent layers are further linked *via* O—H⋯N hydrogen bonding. π–π stacking between the pyridine rings [face-to-face distance = 3.651 (3) Å] occurs in the crystal structure.

## Related literature

For applications of compounds with metal-organic framework structures (MOFs), see: Rowsell & Yaghi (2005 ▶). For the malate ligand, see: Duan *et al.* (2006 ▶); Li *et al.* (2008 ▶); Lin & Xu (2005 ▶); Ou *et al.* (2009 ▶); Xie *et al.* (2004 ▶). For related structures, see: Gadzikwa *et al.* (2008 ▶); Ma *et al.* (2010 ▶); Nordell *et al.* (2003 ▶).
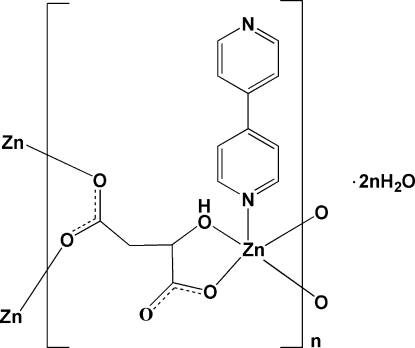


## Experimental

### 

#### Crystal data

[Zn(C_4_H_4_O_5_)(C_10_H_8_N_2_)]·2H_2_O
*M* *_r_* = 389.66
Orthorhombic, 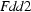

*a* = 17.810 (5) Å
*b* = 47.447 (9) Å
*c* = 7.4063 (15) Å
*V* = 6259 (2) Å^3^
*Z* = 16
Mo *K*α radiation
μ = 1.61 mm^−1^
*T* = 293 K
0.25 × 0.12 × 0.11 mm

#### Data collection

Rigaku R-AXIS RAPID diffractometer
Absorption correction: multi-scan (*ABSCOR*; Higashi, 1995 ▶) *T*_min_ = 0.689, *T*_max_ = 0.843
14679 measured reflections
3380 independent reflections
2817 reflections with *I* > 2σ(*I*)
*R*_int_ = 0.077

#### Refinement

*R*[*F* ^2^ > 2σ(*F* ^2^)] = 0.044
*wR*(*F* ^2^) = 0.087
*S* = 1.09
3380 reflections
217 parameters
1 restraint
H-atom parameters constrained
Δρ_max_ = 0.39 e Å^−3^
Δρ_min_ = −0.37 e Å^−3^
Absolute structure: Flack (1983 ▶), 1438 Friedel pairs
Flack parameter: 0.006 (17)

### 

Data collection: *PROCESS-AUTO* (Rigaku, 1998 ▶); cell refinement: *PROCESS-AUTO*; data reduction: *PROCESS-AUTO*; program(s) used to solve structure: *SHELXS97* (Sheldrick, 2008 ▶); program(s) used to refine structure: *SHELXL97* (Sheldrick, 2008 ▶); molecular graphics: *DIAMOND* (Brandenburg, 1999 ▶); software used to prepare material for publication: *SHELXL97*.

## Supplementary Material

Crystal structure: contains datablock(s) I, global. DOI: 10.1107/S1600536811045788/xu5364sup1.cif

Structure factors: contains datablock(s) I. DOI: 10.1107/S1600536811045788/xu5364Isup2.hkl

Additional supplementary materials:  crystallographic information; 3D view; checkCIF report

## Figures and Tables

**Table 1:** Selected bond lengths (Å)

Zn1—N1	2.066 (3)
Zn1—O1	1.985 (3)
Zn1—O3	2.188 (4)
Zn1—O4^i^	2.031 (4)
Zn1—O5^ii^	1.999 (3)

Symmetry codes: (i) 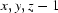; (ii) 
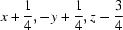
.

**Table 2:** Hydrogen-bond geometry (Å, °)

*D*—H⋯*A*	*D*—H	H⋯*A*	*D*⋯*A*	*D*—H⋯*A*
O1*W*—H11⋯O2^i^	0.86	2.22	2.730 (6)	118
O2*W*—H14⋯O2	0.85	2.43	2.850 (6)	111
O3—H3⋯N2^iii^	0.84	2.13	2.721 (5)	127

Symmetry codes: (i) 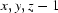; (iii) 
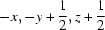
.

**Fig. 1.**
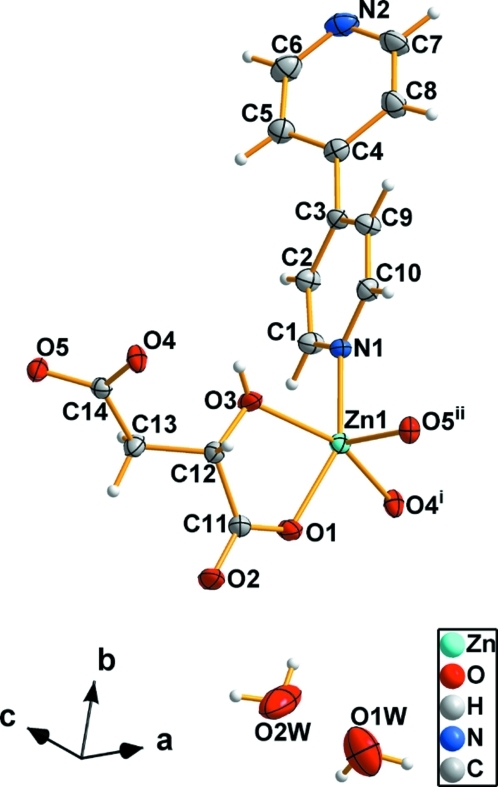
A view of the molecule of title compound with displacement ellipsoids drawn at the 30% probability level. [Symmetry codes: (i) x, y, -1 + z; (ii) 1/4 + x, 1/4 - y, -3/4 + z.]

**Fig. 2.**
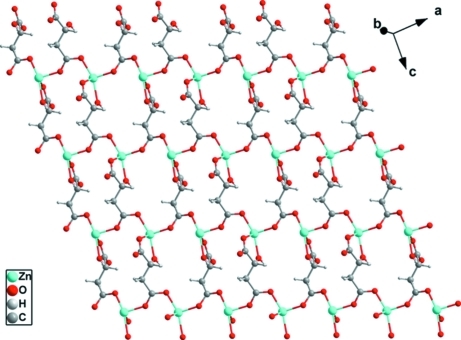
Ball-and-stick representation of the two-dimensional layer structure of title compound. 4,4'-bpy molecule have been omitted for clarity.

**Fig. 3.**
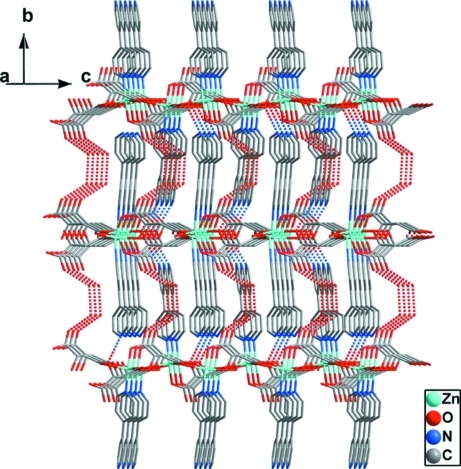
The crystal packing of the title compound along the crystallographic a axis. Interlayer O—H···O(N) hydrogen bonds are shown as dashed lines.

## supplementary crystallographic information

### Comment

The design and synthesis of MOFs with original architectures, which could offer great potential for chemical and structural diversity, is one of the major current challenges in inorganic chemistry (Rowsell & Yaghi, 2005). The malate ligand, besides two terminal carboxyl groups, contains a hydroxyl group in the *α*-position, which can potentially provide an additional coordination site and allows the formation of five- and six-membered rings to stabilize the solid networks (Duan *et al.*, 2006; Xie *et al.*, 2004). In recent years, the construction of MOFs based on malate ligand has been investigated owing to their fascinating coordination modes (Li *et al.*, 2008; Lin *et al.*, 2005; Ou *et al.*, 2009). Herein we report the hydrothermal synthesis and crystal structure of the tile compound.

As shown in Fig. 1, the Zn^II^ ion exhibits a distorted tetragonal pyramid coordination geometry, defined by one N atom from 4,4'-bipyridine molecule, one hydroxyl oxygen and three carboxylate oxygen atoms coming from three different malate ligands. The Zn1—O bond lengths fall in the range of 1.985 (3)–2.188 (4) Å, and the Zn1—N1 distance is 2.066 (3) Å, and the O—Zn—O(N) angles varying from 75.97 (12)–158.80 (11)°, thus falling in the expected region (Gadzikwa *et al.*, 2008). The 4,4'-bipyridine ligand adopts the unidentate coordination mode and the unligated N2 atom acts as the H-bonding acceptor from the hydroxyl group [O3—H3···N2, 2.721 (5) ?] (Ma *et al.*, 2010; Nordell *et al.*, 2003). One carboxylate (O4—C14—O5) of malate dianion act as bidentate bridging and adopt a *µ*_2_-*η*^1^:*η*^1^ coordinated mode, while the carboxyl O1 and the hydroxyl group O3 atoms chelate a Zn ion. The remaining uncoordinated carboxyl O2 atom links with lattice water molecules *via* O—H···O hydrogen bonding (Table 1). As a result, each malate dianion forms four coordination bonds with three Zn centers, leading to a two-dimensional layer structure parallel to the *ac* plane (Fig. 2).

Partially overlapped arrangement is observed between parallel pyridine rings of adjacent layers, the face-to-face separation of 3.644 Å between N1-py and N2^vii^-py rings and 3.435 Å for N2-py and N1^vii^-py rings, indicates the existence of π-π stacking [symmetry code: (vii) -*x*, 1/2 - *y*, 1/2 + *z*]. The adjacent layers are further linked *via* O—H···N(O) hydrogen bonds and *π*···*π* interactions, leading to the formation of a three-dimensional supramolecular framework structure (Table 1 and Fig. 3).

### Experimental

A mixture of Zn(OAc)_2_.2H_2_O (0.22 g, 1.0 mmol), D,L-malic acid (0.27 g, 2.0 mmol), 4,4'-bpy (0.2 g, 1.0 mmol) and 20 ml water was stirred for 2 h in air; it was adjusted to pH = 5.0 with KOH solution (1.0 mol.*L*^-1^) and was heated in a 30 ml stainless steel reactor with a Teflon-liner at 140°C for 3 days, and then cooled to room temperature. Colorless block crystals were isolated with 38% yield (based on Zn). Elemental analysis: Anal. Calcd: C, 43.15; H, 4.11; N, 7.19; Found: C, 42.05; H, 3.97; N, 7.22.

### Refinement

All H atoms were found in a difference Fourier Map and refined as riding with *U*_iso_(H) = 1.5*U*_eq_(O) or *U*_iso_(H) = 1.2*U*_eq_(C).

### Crystal data

**Table:**

[Zn(C_4_H_4_O_5_)(C_10_H_8_N_2_)]·2H_2_O	*F*(000) = 3200
*M**_r_* = 389.66	*D*_x_ = 1.654 Mg m^−^^3^
Orthorhombic, *F**d**d*2	Mo *K*α radiation, λ = 0.71073 Å
Hall symbol: F 2 -2d	Cell parameters from 14444 reflections
*a* = 17.810 (5) Å	θ = 3.0–27.5°
*b* = 47.447 (9) Å	µ = 1.61 mm^−^^1^
*c* = 7.4063 (15) Å	*T* = 293 K
*V* = 6259 (2) Å^3^	Block, colorless
*Z* = 16	0.25 × 0.12 × 0.11 mm

### Data collection

**Table:**

Rigaku R-AXIS RAPID diffractometer	3380 independent reflections
Radiation source: fine-focus sealed tube	2817 reflections with *I* > 2σ(*I*)
graphite	*R*_int_ = 0.077
Detector resolution: 10 pixels mm^-1^	θ_max_ = 27.5°, θ_min_ = 3.0°
ω scans	*h* = −22→22
Absorption correction: multi-scan (*ABSCOR*; Higashi, 1995)	*k* = −60→61
*T*_min_ = 0.689, *T*_max_ = 0.843	*l* = −8→9
14679 measured reflections	

### Refinement

**Table:**

Refinement on *F*^2^	Secondary atom site location: difference Fourier map
Least-squares matrix: full	Hydrogen site location: inferred from neighbouring sites
*R*[*F*^2^ > 2σ(*F*^2^)] = 0.044	H-atom parameters constrained
*wR*(*F*^2^) = 0.087	*w* = 1/[σ^2^(*F*_o_^2^) + (0.0226*P*)^2^ + 22.1041*P*] where *P* = (*F*_o_^2^ + 2*F*_c_^2^)/3
*S* = 1.09	(Δ/σ)_max_ < 0.001
3380 reflections	Δρ_max_ = 0.39 e Å^−^^3^
217 parameters	Δρ_min_ = −0.37 e Å^−^^3^
1 restraint	Absolute structure: Flack (1983), 1438 Friedel pairs
Primary atom site location: structure-invariant direct methods	Flack parameter: 0.006 (17)

### Special details

**Table:**

Geometry. All e.s.d.'s (except the e.s.d. in the dihedral angle between two l.s. planes) are estimated using the full covariance matrix. The cell e.s.d.'s are taken into account individually in the estimation of e.s.d.'s in distances, angles and torsion angles; correlations between e.s.d.'s in cell parameters are only used when they are defined by crystal symmetry. An approximate (isotropic) treatment of cell e.s.d.'s is used for estimating e.s.d.'s involving l.s. planes.
Refinement. Refinement of *F*^2^ against ALL reflections. The weighted *R*-factor *wR* and goodness of fit *S* are based on *F*^2^, conventional *R*-factors *R* are based on *F*, with *F* set to zero for negative *F*^2^. The threshold expression of *F*^2^ > σ(*F*^2^) is used only for calculating *R*-factors(gt) *etc*. and is not relevant to the choice of reflections for refinement. *R*-factors based on *F*^2^ are statistically about twice as large as those based on *F*, and *R*- factors based on ALL data will be even larger.

### Fractional atomic coordinates and isotropic or equivalent isotropic displacement parameters (Å^2^)

**Table:**

	*x*	*y*	*z*	*U*_iso_*/*U*_eq_	
Zn1	0.07684 (2)	0.130827 (9)	0.47984 (7)	0.02744 (12)	
O1	0.0495 (2)	0.09140 (7)	0.5405 (4)	0.0404 (8)	
O2	0.0317 (2)	0.05848 (6)	0.7462 (5)	0.0527 (9)	
O3	0.0542 (2)	0.13206 (6)	0.7701 (5)	0.0485 (10)	
H3	0.0687	0.1404	0.8642	0.073*	
O4	0.06058 (15)	0.12247 (7)	1.2138 (5)	0.0333 (7)	
O5	−0.06183 (15)	0.12117 (6)	1.2620 (4)	0.0342 (8)	
O1W	0.0119 (3)	0.02832 (12)	0.0565 (8)	0.107 (2)	
H11	0.0426	0.0271	−0.0336	0.160*	
H12	−0.0276	0.0221	0.0211	0.160*	
O2W	0.0295 (3)	0.02594 (13)	0.4228 (9)	0.111 (2)	
H14	0.0006	0.0243	0.5139	0.167*	
H13	0.0305	0.0435	0.4020	0.167*	
N1	0.05148 (18)	0.17331 (6)	0.4739 (6)	0.0300 (8)	
N2	−0.0363 (3)	0.31846 (8)	0.4508 (6)	0.0507 (12)	
C1	−0.0156 (2)	0.18258 (9)	0.4188 (6)	0.0364 (11)	
H1	−0.0505	0.1695	0.3778	0.055*	
C2	−0.0353 (2)	0.21050 (9)	0.4200 (6)	0.0344 (10)	
H2	−0.0830	0.2160	0.3828	0.052*	
C3	0.0164 (2)	0.23059 (8)	0.4772 (7)	0.0295 (8)	
C4	−0.0022 (2)	0.26103 (8)	0.4763 (7)	0.0314 (9)	
C5	−0.0736 (3)	0.27109 (9)	0.5116 (7)	0.0395 (12)	
H5	−0.1116	0.2587	0.5455	0.047*	
C6	−0.0880 (3)	0.29946 (10)	0.4965 (9)	0.0501 (13)	
H6	−0.1365	0.3057	0.5193	0.060*	
C7	0.0328 (3)	0.30880 (10)	0.4207 (7)	0.0512 (14)	
H7	0.0700	0.3217	0.3899	0.061*	
C8	0.0521 (3)	0.28080 (10)	0.4329 (7)	0.0433 (12)	
H8	0.1013	0.2752	0.4120	0.052*	
C9	0.0855 (2)	0.22099 (9)	0.5339 (6)	0.0351 (11)	
H9	0.1216	0.2337	0.5735	0.042*	
C10	0.1010 (2)	0.19244 (9)	0.5320 (6)	0.0340 (11)	
H10	0.1476	0.1863	0.5727	0.041*	
C11	0.0415 (2)	0.08348 (9)	0.7015 (7)	0.0331 (10)	
C12	0.0483 (3)	0.10540 (10)	0.8520 (6)	0.0307 (10)	
H4	0.0942	0.1017	0.9209	0.037*	
C13	−0.0179 (2)	0.10388 (9)	0.9781 (7)	0.0347 (9)	
H13A	−0.0317	0.0842	0.9935	0.042*	
H13B	−0.0601	0.1134	0.9217	0.042*	
C14	−0.0056 (2)	0.11663 (8)	1.1619 (5)	0.0271 (9)	

### Atomic displacement parameters (Å^2^)

**Table:**

	*U*^11^	*U*^22^	*U*^33^	*U*^12^	*U*^13^	*U*^23^
Zn1	0.0306 (2)	0.0257 (2)	0.0260 (2)	0.0010 (2)	0.0027 (2)	0.0005 (2)
O1	0.063 (2)	0.0302 (18)	0.0277 (17)	−0.0110 (15)	0.0055 (15)	−0.0037 (13)
O2	0.087 (2)	0.0255 (15)	0.045 (2)	−0.0076 (16)	0.024 (2)	−0.0019 (17)
O3	0.087 (3)	0.0272 (18)	0.032 (2)	−0.0112 (16)	0.0227 (19)	−0.0076 (14)
O4	0.0286 (12)	0.0465 (16)	0.0248 (16)	−0.0048 (14)	0.0006 (16)	0.0045 (16)
O5	0.0312 (14)	0.0401 (16)	0.031 (2)	0.0001 (13)	0.0052 (14)	−0.0037 (14)
O1W	0.108 (4)	0.121 (5)	0.091 (4)	−0.012 (4)	−0.006 (3)	0.054 (3)
O2W	0.092 (4)	0.126 (5)	0.115 (5)	−0.006 (3)	0.004 (3)	−0.073 (4)
N1	0.0365 (18)	0.0225 (17)	0.0311 (19)	−0.0019 (13)	0.006 (2)	−0.0032 (19)
N2	0.079 (3)	0.029 (2)	0.044 (3)	0.012 (2)	−0.005 (3)	0.0018 (19)
C1	0.036 (2)	0.030 (2)	0.043 (3)	−0.0017 (18)	−0.005 (2)	−0.0042 (19)
C2	0.027 (2)	0.032 (2)	0.045 (3)	0.0046 (17)	−0.0037 (19)	−0.0008 (19)
C3	0.036 (2)	0.0235 (19)	0.029 (2)	0.0020 (16)	0.003 (2)	0.004 (2)
C4	0.039 (2)	0.030 (2)	0.025 (2)	0.0064 (18)	−0.005 (2)	0.003 (2)
C5	0.042 (2)	0.036 (2)	0.041 (3)	0.005 (2)	0.000 (2)	0.005 (2)
C6	0.056 (3)	0.043 (3)	0.051 (3)	0.016 (2)	−0.001 (3)	0.002 (3)
C7	0.071 (4)	0.030 (2)	0.053 (3)	−0.008 (2)	0.002 (3)	0.009 (2)
C8	0.045 (3)	0.035 (2)	0.050 (3)	−0.001 (2)	0.004 (2)	0.011 (2)
C9	0.034 (2)	0.029 (2)	0.042 (3)	−0.0037 (19)	−0.004 (2)	−0.0006 (18)
C10	0.030 (2)	0.032 (2)	0.040 (3)	0.0032 (18)	0.0009 (19)	0.0013 (18)
C11	0.036 (2)	0.027 (2)	0.036 (3)	−0.0009 (17)	0.008 (2)	−0.0012 (19)
C12	0.036 (2)	0.028 (2)	0.028 (2)	−0.0003 (18)	0.0056 (19)	−0.0011 (17)
C13	0.033 (2)	0.041 (2)	0.029 (2)	−0.0020 (17)	0.003 (2)	−0.010 (2)
C14	0.033 (2)	0.025 (2)	0.0231 (19)	−0.0028 (17)	−0.0004 (17)	0.0015 (16)

### Geometric parameters (Å, °)

**Table:**

Zn1—N1	2.066 (3)	C2—C3	1.392 (6)
Zn1—O1	1.985 (3)	C2—H2	0.9300
Zn1—O3	2.188 (4)	C3—C9	1.378 (6)
Zn1—O4^i^	2.031 (4)	C3—C4	1.482 (5)
Zn1—O5^ii^	1.999 (3)	C4—C5	1.383 (6)
O1—C11	1.257 (6)	C4—C8	1.385 (6)
O2—C11	1.244 (5)	C5—C6	1.375 (6)
O3—C12	1.407 (5)	C5—H5	0.9300
O3—H3	0.8427	C6—H6	0.9300
O4—C14	1.270 (5)	C7—C8	1.375 (7)
O5—C14	1.265 (5)	C7—H7	0.9300
O1W—H11	0.8645	C8—H8	0.9300
O1W—H12	0.8063	C9—C10	1.383 (6)
O2W—H14	0.8528	C9—H9	0.9300
O2W—H13	0.8466	C10—H10	0.9300
N1—C10	1.337 (5)	C11—C12	1.529 (6)
N1—C1	1.337 (5)	C12—C13	1.506 (6)
N2—C7	1.331 (7)	C12—H4	0.9800
N2—C6	1.333 (6)	C13—C14	1.506 (6)
C1—C2	1.371 (6)	C13—H13A	0.9700
C1—H1	0.9300	C13—H13B	0.9700
			
O1—Zn1—O5^ii^	99.87 (13)	C6—C5—H5	120.2
O1—Zn1—O4^i^	90.04 (13)	C4—C5—H5	120.2
O5^ii^—Zn1—O4^i^	104.34 (12)	N2—C6—C5	123.7 (5)
O1—Zn1—N1	150.56 (13)	N2—C6—H6	118.2
O5^ii^—Zn1—N1	105.40 (12)	C5—C6—H6	118.2
O4^i^—Zn1—N1	97.97 (16)	N2—C7—C8	123.6 (5)
O1—Zn1—O3	75.97 (12)	N2—C7—H7	118.2
O5^ii^—Zn1—O3	93.84 (15)	C8—C7—H7	118.2
O4^i^—Zn1—O3	158.80 (11)	C7—C8—C4	119.7 (5)
N1—Zn1—O3	87.40 (15)	C7—C8—H8	120.2
C11—O1—Zn1	121.6 (3)	C4—C8—H8	120.2
C12—O3—Zn1	114.4 (3)	C3—C9—C10	120.0 (4)
C12—O3—H3	95.2	C3—C9—H9	120.0
Zn1—O3—H3	140.2	C10—C9—H9	120.0
C14—O4—Zn1^iii^	117.9 (3)	N1—C10—C9	122.5 (4)
C14—O5—Zn1^iv^	135.0 (3)	N1—C10—H10	118.8
H11—O1W—H12	106.0	C9—C10—H10	118.8
H14—O2W—H13	104.2	O2—C11—O1	123.6 (4)
C10—N1—C1	117.7 (4)	O2—C11—C12	117.7 (4)
C10—N1—Zn1	120.8 (3)	O1—C11—C12	118.6 (4)
C1—N1—Zn1	121.5 (3)	O3—C12—C13	111.7 (4)
C7—N2—C6	116.6 (4)	O3—C12—C11	107.6 (4)
N1—C1—C2	123.0 (4)	C13—C12—C11	111.0 (4)
N1—C1—H1	118.5	O3—C12—H4	108.8
C2—C1—H1	118.5	C13—C12—H4	108.8
C1—C2—C3	119.6 (4)	C11—C12—H4	108.8
C1—C2—H2	120.2	C12—C13—C14	115.3 (3)
C3—C2—H2	120.2	C12—C13—H13A	108.5
C9—C3—C2	117.2 (4)	C14—C13—H13A	108.5
C9—C3—C4	121.5 (4)	C12—C13—H13B	108.5
C2—C3—C4	121.2 (4)	C14—C13—H13B	108.5
C5—C4—C8	116.8 (4)	H13A—C13—H13B	107.5
C5—C4—C3	122.7 (4)	O5—C14—O4	121.4 (4)
C8—C4—C3	120.4 (4)	O5—C14—C13	118.8 (3)
C6—C5—C4	119.6 (5)	O4—C14—C13	119.8 (4)

Symmetry codes: (i) *x*, *y*, *z*−1; (ii) *x*+1/4, −*y*+1/4, *z*−3/4; (iii) *x*, *y*, *z*+1; (iv) *x*−1/4, −*y*+1/4, *z*+3/4.

### Hydrogen-bond geometry (Å, °)

**Table:**

*D*—H···*A*	*D*—H	H···*A*	*D*···*A*	*D*—H···*A*
O1W—H11···O2^i^	0.86	2.22	2.730 (6)	118.
O2W—H14···O2	0.85	2.43	2.850 (6)	111.
O3—H3···N2^v^	0.84	2.13	2.721 (5)	127.

Symmetry codes: (i) *x*, *y*, *z*−1; (v) −*x*, −*y*+1/2, *z*+1/2.

## References

[bb1] Brandenburg, K. (1999). *DIAMOND.* Crystal Impact GbR, Bonn, Germany.

[bb2] Duan, L.-M., Xie, F.-T., Chen, X.-Y., Chen, Y., Lu, Y.-K., Cheng, P. & Xu, J.-Q. (2006). *Cryst. Growth Des.* **5**, 1101–1106.

[bb3] Flack, H. D. (1983). *Acta Cryst.* A**39**, 876–881.

[bb4] Gadzikwa, T., Zeng, B. S., Hupp, J. T. & Nguyen, S. T. (2008). *Chem. Commun.* pp. 3672–3674. 10.1039/b714160b 18665295

[bb5] Higashi, T. (1995). *ABSCOR.* Rigaku Corporation, Tokyo, Japan.

[bb6] Li, J.-H., Nie, J.-J., Su, J.-R. & Xu, D.-J. (2008). *Acta Cryst.* E**64**, m538–m539. 10.1107/S1600536808006715 PMC2960982 21201998

[bb7] Lin, D.-D. & Xu, D.-J. (2005). *Acta Cryst.* E**61**, m1215–m1217.

[bb8] Ma, L.-F., Meng, Q.-L., Li, C.-P., Li, B., Wang, L.-Y., Du, M. & Liang, F.-P. (2010). *Cryst. Growth Des.* **10**, 3036–3043.

[bb9] Nordell, K. J., Higgins, K. A. & Smith, M. D. (2003). *Acta Cryst.* E**59**, m114–m115.

[bb10] Ou, G.-C., Zhou, Q. & Ng, S. W. (2009). *Acta Cryst.* E**65**, m728. 10.1107/S1600536809020662 PMC2969409 21582671

[bb11] Rigaku (1998). *PROCESS-AUTO* Rigaku Corporation, Tokyo, Japan.

[bb12] Rowsell, J. L. C. & Yaghi, O. M. (2005). *Angew. Chem. Int. Ed.* **44**, 4670–4679. 10.1002/anie.200462786 16028207

[bb13] Sheldrick, G. M. (2008). *Acta Cryst.* A**64**, 112–122. 10.1107/S0108767307043930 18156677

[bb14] Xie, F.-T., Duan, L.-M., Xu, J.-Q., Ye, L., Liu, Y.-B. & Hu, X.-X. (2004). *Eur. J. Inorg. Chem.* pp. 4375–4379.

